# 4408 Using a human-centered design process to address challenges of engaging pregnant & parenting women with opioid use disorder

**DOI:** 10.1017/cts.2020.284

**Published:** 2020-07-29

**Authors:** Sarah Wiehe, Dustin Lynch, Courtney Moore, Brandon Cockrum, Bridget Hawryluk, Gina Claxton

**Affiliations:** 1 Indiana University School of Medicine; 2 Indiana Clinical and Translational Sciences Institute

## Abstract

OBJECTIVES/GOALS: Using a human-centered approach, IDEO, a nationally-renown human-centered design team, and Research Jam, Indiana CTSI’s patient engagement core, integrated and tailored complimentary programs to address the challenges of engaging mothers with opioid misuse around the time of birth. METHODS/STUDY POPULATION: Gathered data through focus groups, site visits, and one-on-one interviews with key stakeholders: mothers in opioid use recovery, peer recovery coaches, and other people living with or directly affected by opioid use disorder (OUD). RESULTS/ANTICIPATED RESULTS: Themes emerged around stigma (e.g., constant judgment, majority of interactions focused on addiction, addiction comes from bad choices), the healthcare system (e.g., healthcare system bias and stigma, misalignment of services and timing of need, no support for support network), and relating to recovery (very variable but generally ambiguous and uncertain process and outcomes, importance of peer recovery coaches, importance of community resources). Identified themes were used to create insights that informed the underlying concepts of an engagement strategy including support and resources for recovery coaches, and education materials for mothers with OUD. One of human-centered design’s strengths is iteration, and the materials created for this have yet to be tested and refined thoroughly to be meaningful and lasting interventions. DISCUSSION/SIGNIFICANCE OF IMPACT: Considerable insights into the lived experience of those experiencing OUD and those who support these individuals yielded tangible ways to test improved engagement and recruitment of women with OUD at the time of birth.

